# 1185. Evaluation of Antimicrobial Stewardship Practices at a Veterans Affairs Health Care System Community Living Center

**DOI:** 10.1093/ofid/ofad500.1025

**Published:** 2023-11-27

**Authors:** Andrea Ampuero, Brittany Melville, Bailey Guest, Joseph Creel

**Affiliations:** Fayetteville VA Health Care System, Fayetteville, North Carolina; Salisbury VA Health Care System, Salisbury, North Carolina; Salisbury VA Health Care System, Salisbury, North Carolina; Atrium Health Wake Forest Baptist, Winston-Salem, North Carolina

## Abstract

**Background:**

Antimicrobial stewardship programs (ASP) have demonstrated improvement in patient outcomes, reduction of antimicrobial adverse drug events (ADE) and a decrease in antimicrobial resistance in hospitals. Antimicrobials maintain the second highest rate of ADEs in long-term care facilities. Prescribed antimicrobials in nursing homes may be unnecessary or inappropriate in upwards of 75% of cases. There is limited evidence available quantifying the impact of pharmacist-led ASP interventions in long-term care facilities. The purpose of this project is to evaluate the effectiveness of antimicrobial stewardship interventions completed at the Salisbury Veterans Affairs Health Care System Community Living Center (SVAHCS CLC).

**Methods:**

This is a retrospective quality improvement project. Eligible subjects included Veterans residing in the SVAHCS CLC to which an ASP intervention documented in TheraDoc^®^ was proposed from May 2018 to January 2021. The primary objective is to determine the effectiveness of ASP interventions in the CLC based on acceptance rate. Secondary objectives include to assess safety of select implemented CLC ASP interventions through reported ADEs within 30 days of performed CLC ASP intervention and determine potential cost savings of the implemented ASP interventions.

**Results:**

A total of 379 interventions were included. Of these interventions, 370 were accepted (98%), 5 were accepted with modification (1%) and 4 were rejected (1%). The most common ASP interventions included lab monitoring, renal adjustment, pharmacokinetic consults and duration changes. The most common indications for many ASP interventions performed were osteomyelitis, influenza and bacteremia. Vancomycin, cefepime and ceftriaxone were the most common antimicrobials involved for which ASP interventions were performed. Of the 131 interventions assessed for safety, one Veteran experienced ADEs within 30 days of the intervention including nephrotoxicity and *Clostridioides difficile* infection. There was a total potential cost savings of $101,704.

Most Common Infections

**Figure:**
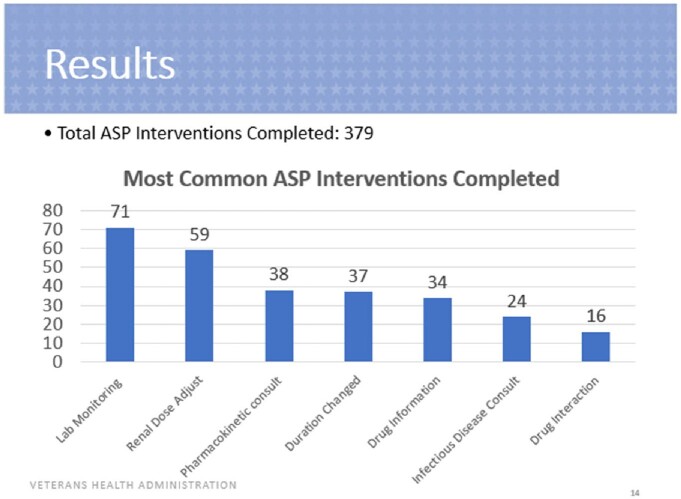

ASP Intervention Acceptance Rate and ADEs within 30 Days of ASP Intervention

**Figure:**
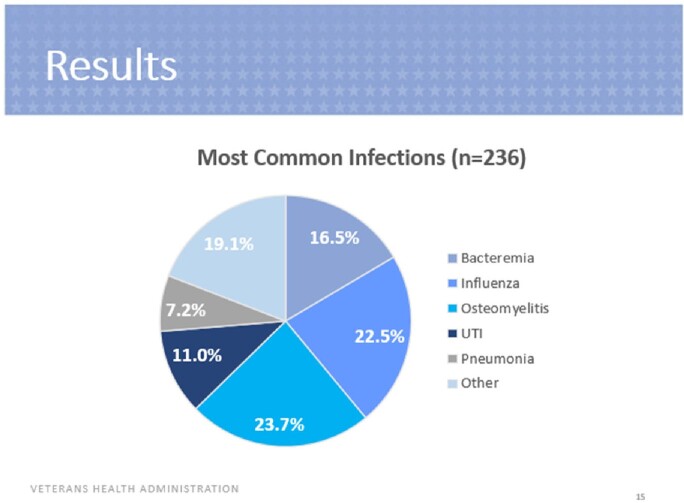

**Figure:**
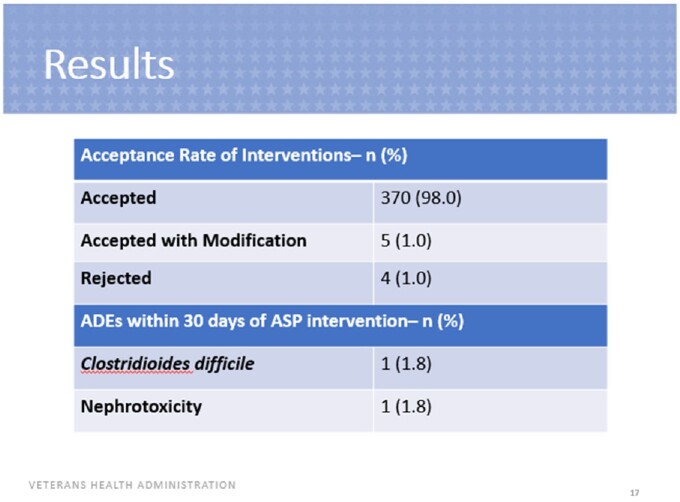

**Conclusion:**

This study demonstrates that pharmacist-led ASP interventions proposed in the SVAHCS CLC were effective with a high rate of acceptance. These interventions resulted in a low rate of ADEs and potential cost savings for the facility.

**Disclosures:**

**All Authors**: No reported disclosures

